# Supplementary material for: Preferential Ribosome Loading on the Stress-Upregulated mRNA Pool Shapes the Selective Translation under Stress Conditions
Source: Plants (Basel). 2021 Feb 5;10(2):304. doi: 10.3390/plants10020304 (PMC7915710; doi:10.3390/plants10020304)
Supplement: Supplementary file 1 [file plants-10-00304-s001.zip › plants-1072824-supplementary/SupFigure.pdf]

Figure S1

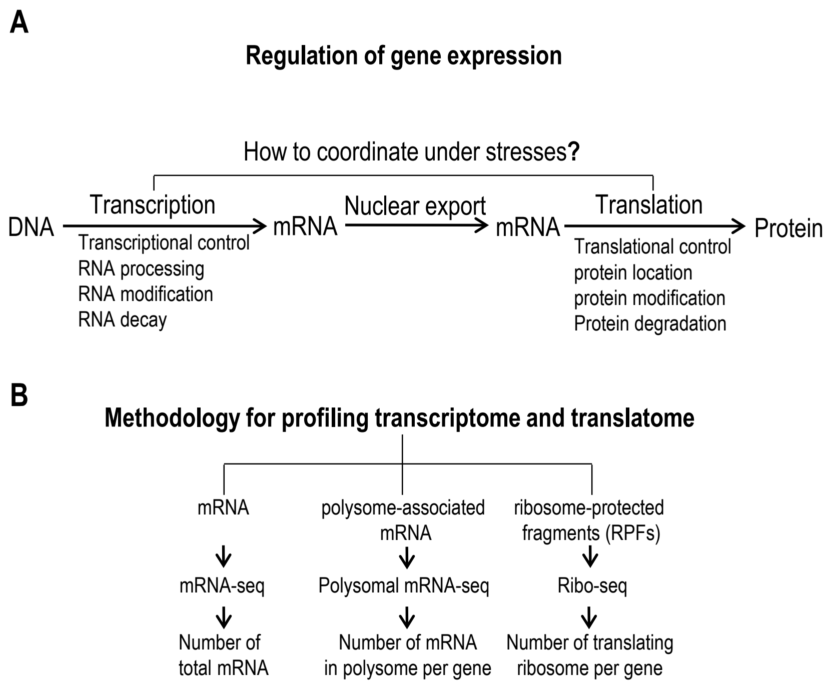

Figure S1. Schematic of gene expression regulation and related methodologies.

Figure S2

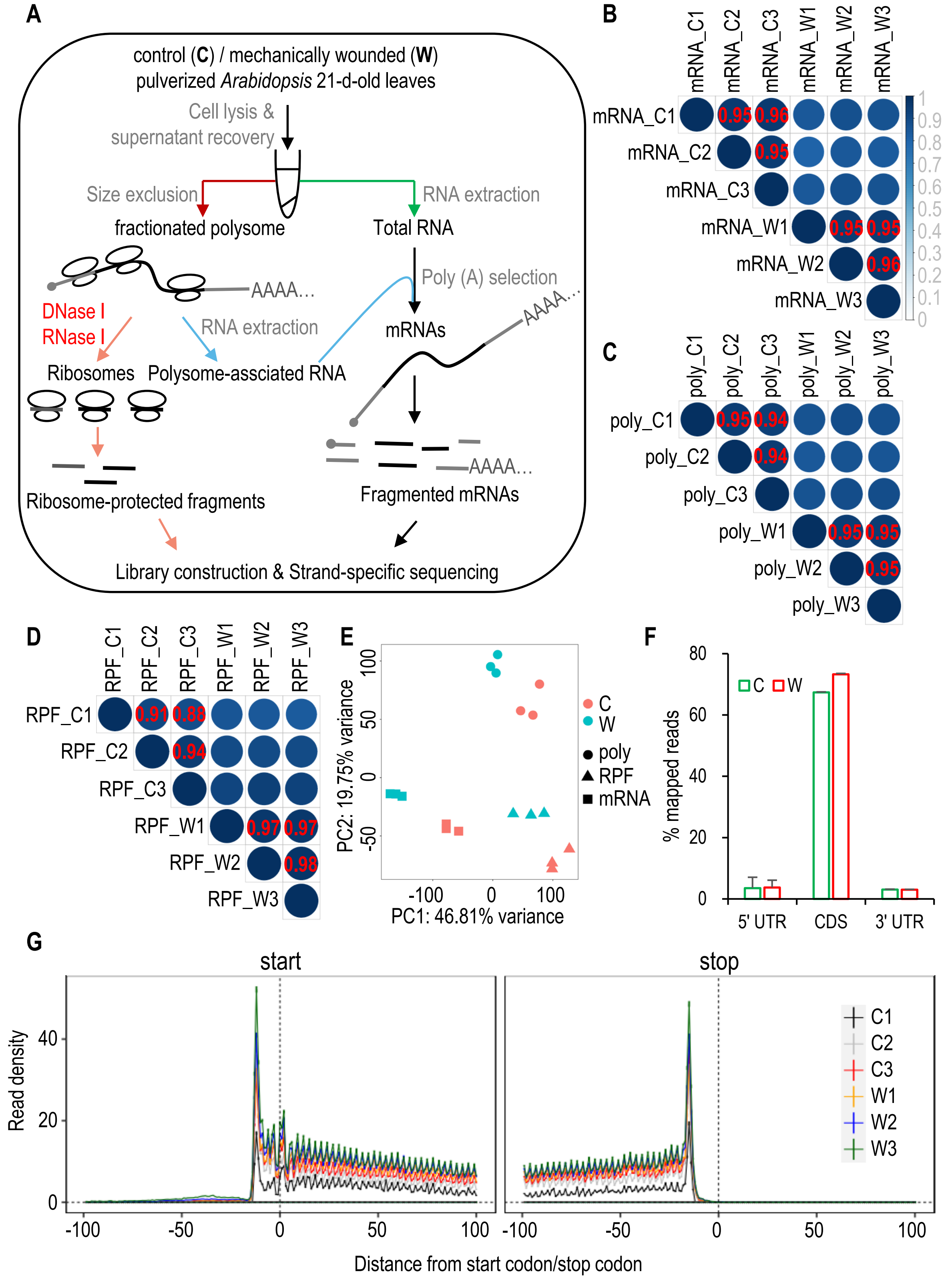

A
control (C) / mechanically wounded (W)
pulverized Arabidopsis 21-d-old leaves
Cell lysis &
supernatant recovery
Size exclusion
RNA extraction
fractionated polysome
Total RNA
AAAA...
Poly (A) selection
DNase I
RNase I
RNA extraction
mRNAs
AAAA...
Ribosomes
Polysome-assciated RNA
AAAA...
Ribosome-protected fragments
Fragmented mRNAs
Library construction & Strand-specific sequencing
B
mRNA_C1
mRNA_C2
mRNA_C3
mRNA_W1
mRNA_W2
mRNA_W3
0.95 0.96
0.95
0.95 0.95
0.96
C
poly_C1
poly_C2
poly_C3
poly_W1
poly_W2
poly_W3
0.95 0.94
0.94
0.95 0.95
0.95
D
RPF_C1
RPF_C2
RPF_C3
RPF_W1
RPF_W2
RPF_W3
0.91 0.88
0.94
0.97 0.97
0.98
E
PC2: 19.75% variance
PC1: 46.81% variance
C
W
poly
RPF
mRNA
F
% mapped reads
C W
5' UTR
CDS
3' UTR
G
start
stop
Read density
Distance from start codon/stop codon
C1
C2
C3
W1
W2
W3

Figure S2

Figure S2. Schematic representation of experimental design and the characteristics of ribosome profiling data from control and wounded Arabidopsis leaves. (A) Overview of experimental strategy. Total mRNA-seq, polysome associated mRNA-seq and ribosome profiling were performed with C (control) and W (wounded) leaves. In total, ~18 and ~41 million (M) uniquely mapped reads were obtained from ribosome profiling libraries for the control (C1 2.33 M, C2 5.38 M, C3 10.54 M) and wounded (W1 11.30 M, W2 15.02 M, W3 15.14 M) samples, respectively. Likewise, ~16 and ~17 M from total mRNA-seq libraries (C1 4.27 M, C2 7.58 M, C3 5.01 M, W1 4.14 M, W2 8.43 M, W3 5.20 M); ~28 and ~14 M from polysome associated mRNA-seq libraries (C1 11.89 M, C2 11.37 M, C3 5.27 M, W1 5.18 M, W2 4.09 M, W3 4.83 M). (B-D) High correlation across three biological replicates of the total mRNAs (mRNA), polysome associated mRNAs (poly) and ribosome-protected fragments (RPF) sequencing libraries with or without wounding. The base-2 logarithm (log_2) of the Transcripts Per Million (TPM) was used to calculate the Pearson correlation coefficient between samples; The colored number is the Pearson correlation coefficient between two samples. (E) Principal component analysis (PCA) of three biological replicates from total mRNAs (mRNA), polysome-associated mRNAs (poly) and ribosome-protected fragments (RPF) before and after wounding. (F) Percentages of RPFs assigned to the CDS, 5' UTR and 3' UTR in C and W samples. (G) Read density of the RPFs 5'-end around the annotated start or stop codon (100-nt upstream and downstream).

Figure S3

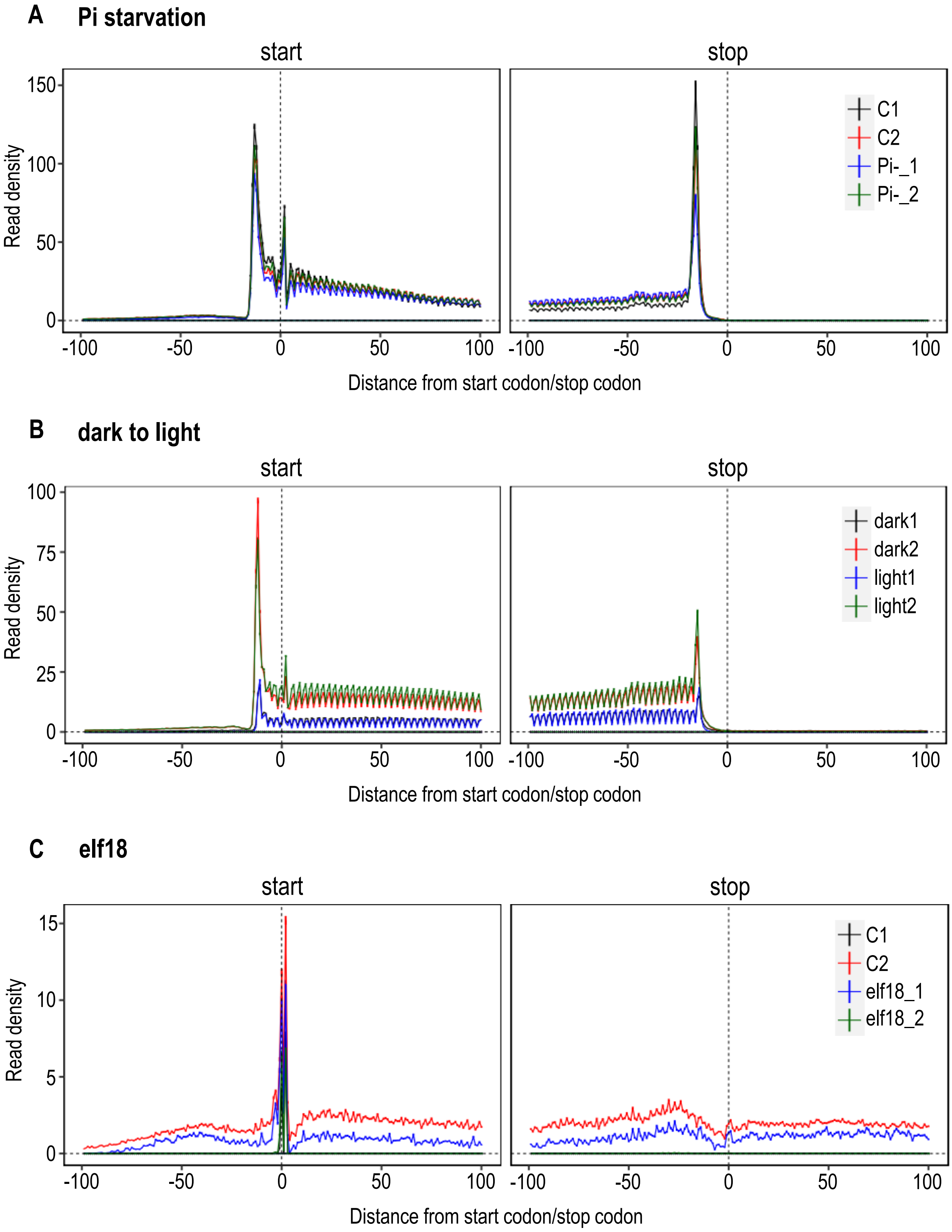
A
Pi starvation
start
stop
Read density
150
100
50
0
-100
-50
0
50
100
Distance from start codon/stop codon
C1
C2
Pi-_1
Pi-_2
B
dark to light
start
stop
Read density
100
75
50
25
0
dark1
dark2
light1
light2
Distance from start codon/stop codon
C
elf18
start
stop
Read density
15
10
5
0
C1
C2
elf18_1
elf18_2
Distance from start codon/stop codon

Figure S3

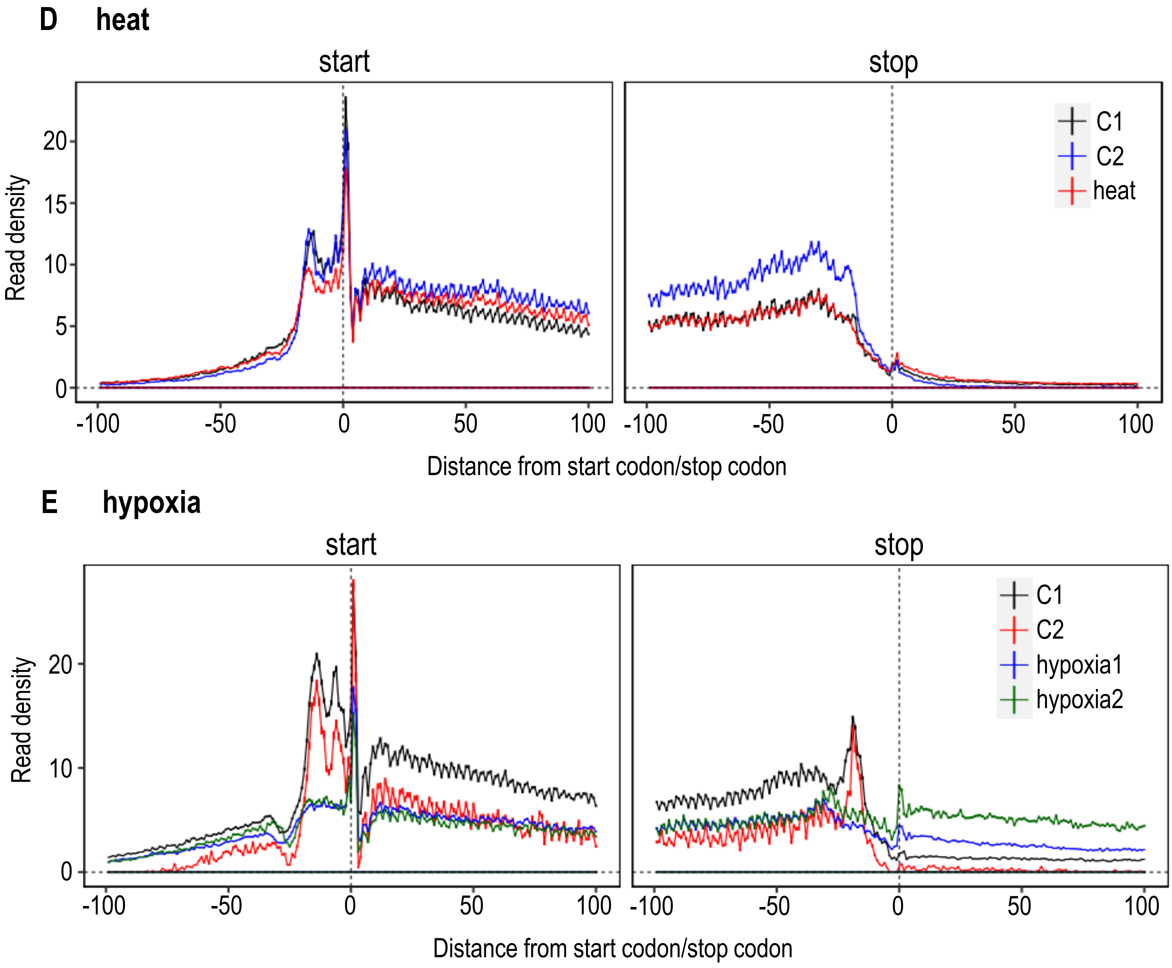

Figure S3. Read density of the RPFs 5'-end around the annotated start or stop codon (100-nt upstream and downstream) under different stresses: (A) Pi starvation, (B) dark to light, (C) elf18, (D) heat and (E) hypoxia.

Figure S4

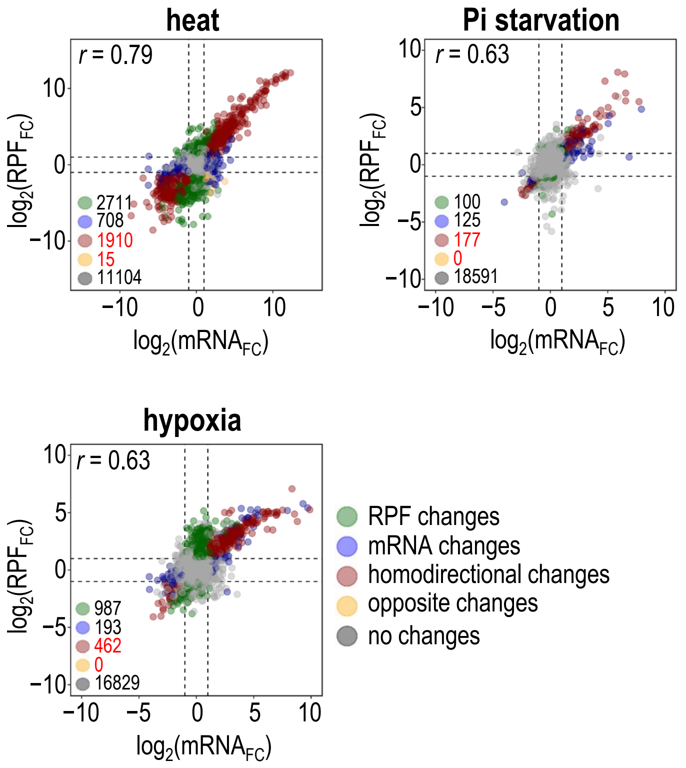

Figure S4. Correlations of $mRNA_{FC}$ and RPF_{FC} introduced by indicated stresses. Detailed description as in Figure 1B.

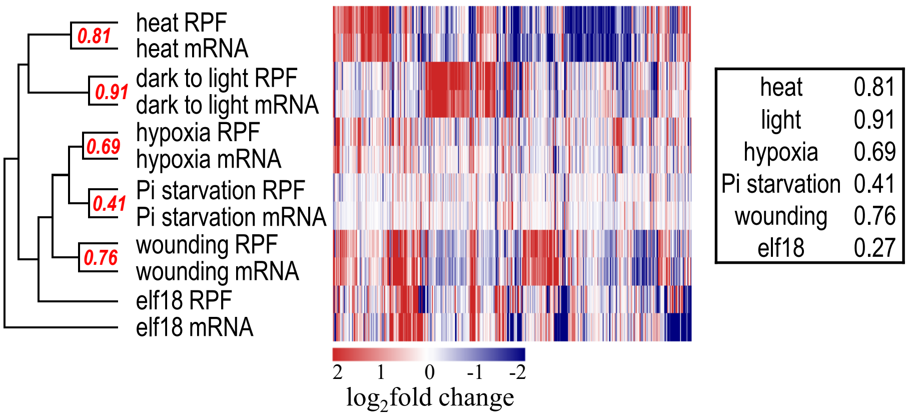

Figure S5. Relationships between $mRNA_{FC}$ and RPF_{FC} under different stresses. Hierarchical cluster analysis was conducted on 7456 genes, which are differentially expressed at both mRNA and RPF levels under at least one of the indicated stresses. Each column represents one gene and each row represents one experimental dataset. log_2FC of each gene at mRNA and/or RPF levels under each stress are color coded with a bar at the bottom. Pearson correlations of $mRNA_{FC}$ and RPF_{FC} upon each stress are visualized with a tree on the left and a table on the right.

Figure S6

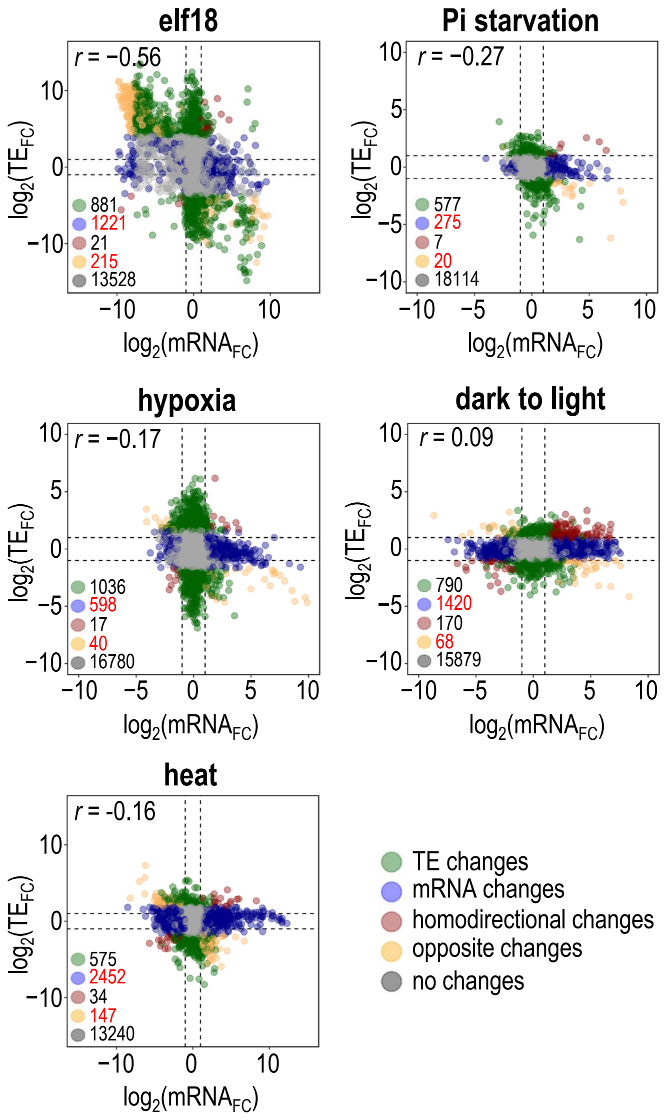

Figure S6. Correlations of $mRNA_{FC}$ and TE_{FC} introduced by indicated stresses. Detailed description as in Figure 1F.

Figure S7

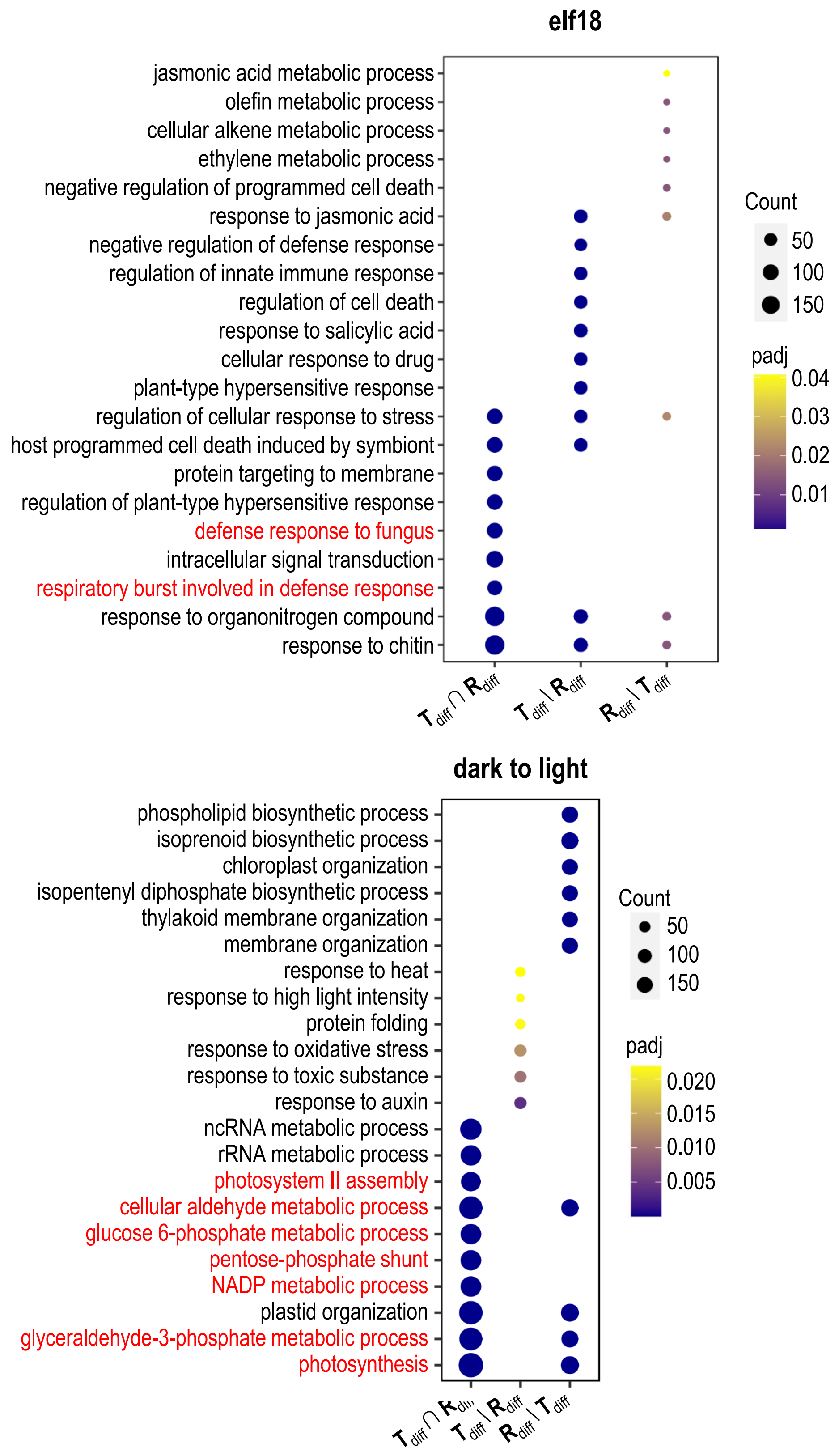

elf18
jasmonic acid metabolic process
olefin metabolic process
cellular alkene metabolic process
ethylene metabolic process
negative regulation of programmed cell death
response to jasmonic acid
negative regulation of defense response
regulation of innate immune response
regulation of cell death
response to salicylic acid
cellular response to drug
plant-type hypersensitive response
regulation of cellular response to stress
host programmed cell death induced by symbiont
protein targeting to membrane
regulation of plant-type hypersensitive response
defense response to fungus
intracellular signal transduction
respiratory burst involved in defense response
response to organonitrogen compound
response to chitin
$T_{diff} \cap R_{diff}$
$T_{diff} \setminus R_{diff}$
$R_{diff} \setminus T_{diff}$
Count
50
100
150
padj
0.04
0.03
0.02
0.01
dark to light
phospholipid biosynthetic process
isoprenoid biosynthetic process
chloroplast organization
isopentenyl diphosphate biosynthetic process
thylakoid membrane organization
membrane organization
response to heat
response to high light intensity
protein folding
response to oxidative stress
response to toxic substance
response to auxin
ncRNA metabolic process
rRNA metabolic process
photosystem II assembly
cellular aldehyde metabolic process
glucose 6-phosphate metabolic process
pentose-phosphate shunt
NADP metabolic process
plastid organization
glyceraldehyde-3-phosphate metabolic process
photosynthesis
$T_{diff} \cap R_{diff}$
$T_{diff} \setminus R_{diff}$
$R_{diff} \setminus T_{diff}$
Count
50
100
150
padj
0.020
0.015
0.010
0.005

Figure S7

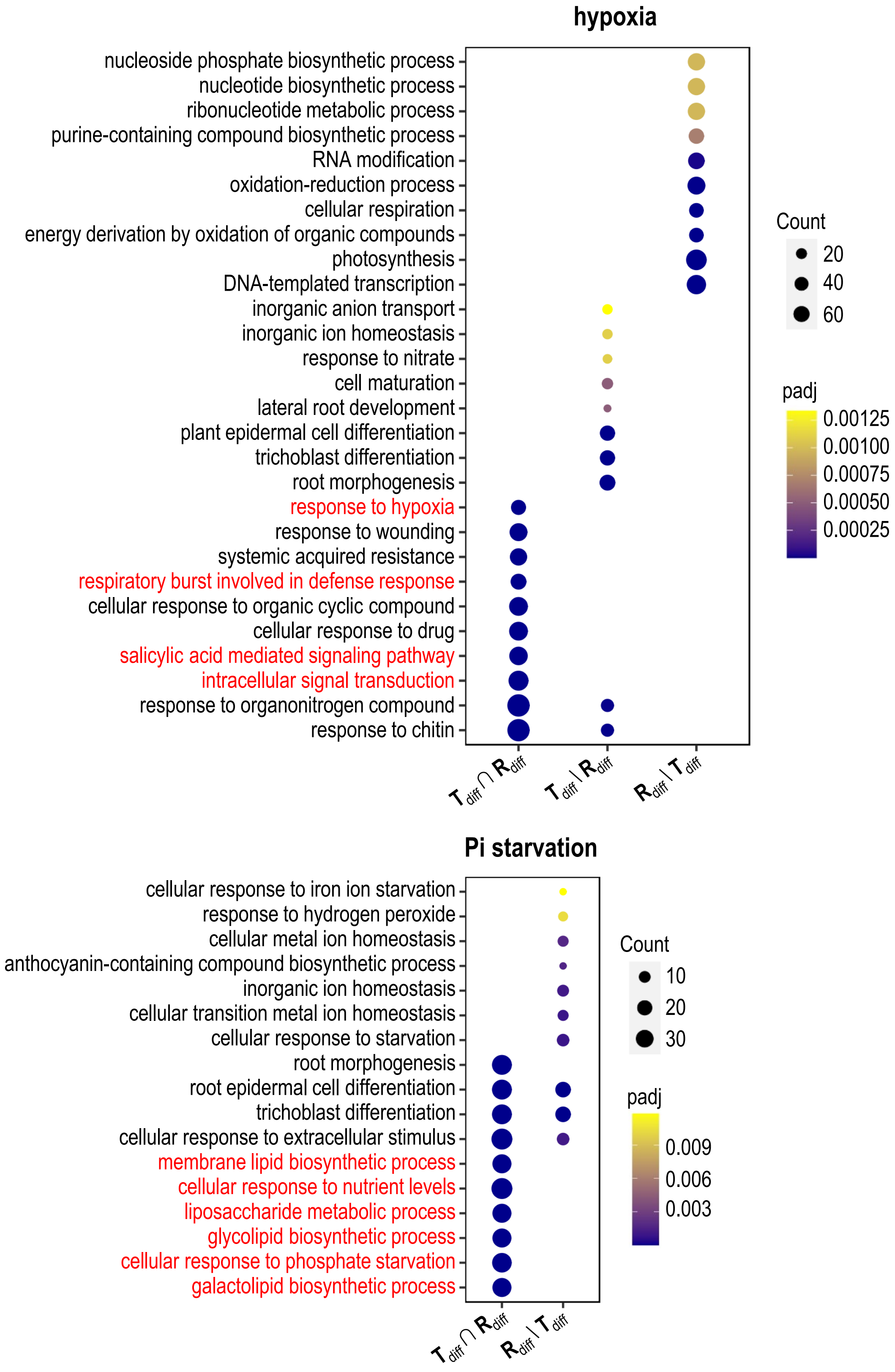

hypoxia
nucleoside phosphate biosynthetic process
nucleotide biosynthetic process
ribonucleotide metabolic process
purine-containing compound biosynthetic process
RNA modification
oxidation-reduction process
cellular respiration
energy derivation by oxidation of organic compounds
photosynthesis
DNA-templated transcription
inorganic anion transport
inorganic ion homeostasis
response to nitrate
cell maturation
lateral root development
plant epidermal cell differentiation
trichoblast differentiation
root morphogenesis
response to hypoxia
response to wounding
systemic acquired resistance
respiratory burst involved in defense response
cellular response to organic cyclic compound
cellular response to drug
salicylic acid mediated signaling pathway
intracellular signal transduction
response to organonitrogen compound
response to chitin
$T_{diff} \cap R_{diff}$
$T_{diff} \setminus R_{diff}$
$R_{diff} \setminus T_{diff}$
Count
20
40
60
padj
0.00125
0.00100
0.00075
0.00050
0.00025
Pi starvation
cellular response to iron ion starvation
response to hydrogen peroxide
cellular metal ion homeostasis
anthocyanin-containing compound biosynthetic process
inorganic ion homeostasis
cellular transition metal ion homeostasis
cellular response to starvation
root morphogenesis
root epidermal cell differentiation
trichoblast differentiation
cellular response to extracellular stimulus
membrane lipid biosynthetic process
cellular response to nutrient levels
liposaccharide metabolic process
glycolipid biosynthetic process
cellular response to phosphate starvation
galactolipid biosynthetic process
$T_{diff} \cap R_{diff}$
$R_{diff} \setminus T_{diff}$
Count
10
20
30
padj
0.009
0.006
0.003

Figure S7

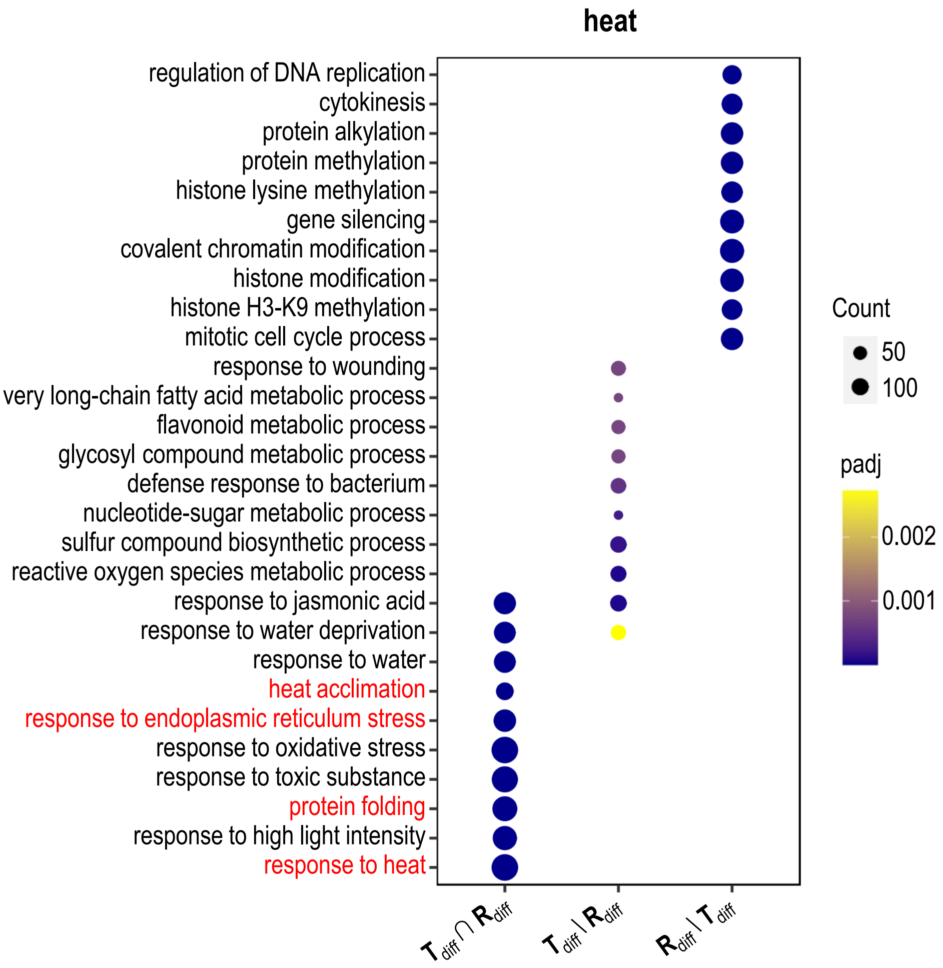

Figure S7. Functional categories of differentially expressed genes under different stresses. Detailed description as in Figure 3B.

Figure S8

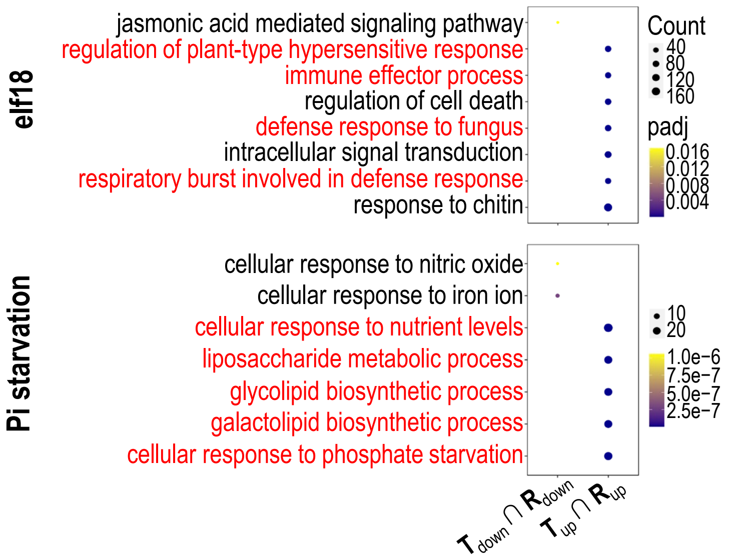

Figure S8. Functional categories of up- or down- regulated genes in response to elf18 or Pi starvation. $T_{up} \cap R_{up}$ represents genes up-regulated at both transcriptional (mRNA) and translational (RPF) levels; $T_{down} \cap R_{down}$ represents genes down-regulated at both transcriptional and translational levels.
