# Supplementary material for: Preferential Ribosome Loading on the Stress-Upregulated mRNA Pool Shapes the Selective Translation under Stress Conditions
Source: Plants (Basel). 2021 Feb 5;10(2):304. doi: 10.3390/plants10020304 (PMC7915710; doi:10.3390/plants10020304)
Supplement: Supplementary file 1 [file plants-10-00304-s001.zip › plants-1072824-supplementary/Supplemental Methods.pdf]

Detailed description and code for the bioinformatic analysis:

1. Acquisition of the public datasets used in this study:

In this study, we used the published datasets from five studies interpreting different stress responses in Arabidopsis, including dark to light transition, heat, hypoxia, Pi starvation and the pathogen-associated molecular pattern (elf18). The list of all the public datasets used in this study and the code for data acquisition are as below:

(1) Public datasets used in this study:

Dark to light transition:

GSM1068665 mRNASS-Dark
GSM1068666 mRNASS-L4h
GSM1068667 mRNARP-Dark
GSM1068668 mRNARP-L4h
GSM1226366 mRNASS-Dark_2
GSM1226367 mRNASS-L4h_2
GSM1226368 mRNARP-Dark_2
GSM1226369 mRNARP-L4h_2

Heat:

GSM1709060 control RNA
GSM1709061 control RPF
GSM1709062 control RNA biological replicate
GSM1709063 control RPF biological replicate
GSM1709064 heat stress RNA
GSM1709065 heat stress RPF

Hypoxia:

GSM1224473 RS_Stress1
GSM1224474 RS_Stress3
GSM1224475 RS_NoStress2
GSM1224476 RS_NoStress3
GSM1224479 Total_no_stress1
GSM1224480 Total_stress1
GSM1224483 Total_no_stress2
GSM1224484 Total_stress2

Pi starvation:

GSM2601708 RS_NoStress (two replicates)
GSM2601709 RS_P_Stress (two replicates)
GSM2601710 Total_NoStress (two replicates)
GSM2601711 Total_P_stress (two replicates)

Pathogen-associated molecular pattern (elf18):

GSM2306286 RS-Mock Rep1
GSM2306287 RS-Mock Rep2
GSM2306288 RS-elf18 Rep1
GSM2306289 RS-elf18 Rep2
GSM2306290 RF-Mock Rep1
GSM2306291 RF-Mock Rep2
GSM2306292 RF-elf18 Rep1
GSM2306293 RF-elf18 Rep2

(2) Data acquisition:

```
a="cat SraAccList.txt" # SraAccList.txt is the Sequence Read Archive (SRA) accession list
for i in $a;do prefetch -O ./ $i;done
```

(3) From SRA to fastq:

```
# using the fastq-dump command to convert the SRA file to fastq
fastq-dump --split-3 accession.sra
```

2. **Quality assessment using the software FASTQC**

```
find *.fastq | xargs fastqc -t 10 # * represents the filename
```

3. **Adaptor removal by cutadapt (version 2.4)**

all the public datasets were generated by single end sequencing, and our self-produced data were obtained through pair end sequencing

* represents the filename

reads shorter than 20 nt were removed through the parameter -m 20.

# Dark to light transition:

```
cutadapt -a CTGTAGGCACCATCAAT -z -O 5 -e 0.1 -m 20 -q 20 -o *clean.fastq *.fastq
```

# Heat:

```
cutadapt -a TGGAATTCTC -z -O 5 -e 0.1 -m 20 -q 20 -o *clean.fastq *.fastq
```

# Hypoxia:

```
cutadapt -a TGGAATTCTC -z -O 5 -e 0.1 -m 20 -q 20 -o *RNA.clean.fastq *RNA.fastq
cutadapt -a AAAAAAAAAAAA -z -O 5 -e 0.1 -m 20 -q 20 -o *RPF.clean.fastq *RPF.fastq
```

# Pi starvation:

```
cutadapt -a CTGTAGGCACCATCAATAGATCGGAAGAG -z -O 5 -e 0.1 -m 20 -q 20 -o
*RNA.clean.fastq *RNA.fastq
cutadapt -a AGATCGGAAGAGCACACGTCTGAACTCCAGTCA -z -O 5 -e 0.1 -m 20 -q 20 -o
*RPF.clean.fastq *RPF.fastq
```

# Pathogen-associated molecular pattern (elf18):

```
cutadapt -a CTGTAGGCACCATCAAT -z -O 5 -e 0.1 -m 20 -q 20 -o *clean.fastq *.fastq
```

Wounding (self-produced data):**

cutadapt -a TGGAATTCTCGGGTG -A GATCGTCGGACTGTA -z -e 0.1 -O 5 -m 20 -q 20 -o *RPF.clean.R1.fq.gz -p *RPF.clean.R2.fq.gz *RPF.R1.fastq.gz *RPF.R2.fastq.gz

cutadapt -a AGATCGGAAGAGCACACGT -A AGATCGGAAGAGCACACGT -z -O 5 -e 0.1 -m 20 -q 20 -o *total (poly).clean.R1.fq.gz -p total (poly).clean.R2.fq.gz *total (poly).R1.fastq.gz *total (poly).R2.fastq.gz

4. **Align clean reads to the TAIR10 genome using STAR (version 2.7.4a)**

Dark to light transition/Heat/Hypoxia/Pi starvation/elf18:

single-end mapping (for public data)

STAR --genomeDir /public/genome/arabidopsis/tair10/star-index --runThreadN 16 --readFilesIn *clean.fastq --outFileNamePrefix ./*_ --outSAMtype BAM SortedByCoordinate --outFilterMismatchNmax 2 --outFilterMultimapNmax 1

pair-end mapping (for self-produced wounding data):

STAR --genomeDir /public/genome/arabidopsis/tair10/star-index --runThreadN 16 --readFilesCommand zcat --readFilesIn *clean_R1.fq.gz *clean_R2.fq.gz --outFileNamePrefix ./*_ --outSAMtype BAM SortedByCoordinate --outFilterMismatchNmax 2 --outFilterMultimapNmax 1

5. **Read assignment to genomic features using featureCounts (version 1.6.5)**

# Read assignment to exons:

input data is the uniquely mapped reads (*.bam)

read counts along exons were used for differential expression analysis

/software/subread-1.6.5-Linux-x86_64/bin/featureCounts -T 16 -t exon -g gene_id -s 1 -a araport11.gtf -o *count.txt *.bam

# Read assignment to CDS / 5' UTR / 3' UTR:

input data is the uniquely mapped reads (*.bam)

this analysis is only conducted on our self-produced Ribo-seq data

/software/subread-1.6.5-Linux-x86_64/bin/featureCounts -T 16 -t CDS/ five_prime_utr/three_prime_utr -g gene_id -s 1 -a araport11.gtf -o *count.txt *.bam

6. **Visualization of the read coverage via Integrative Genomics Viewer (IGV)**

samtools sort *.bam > *.sort.bam

samtools index *.sort.bam

bamCoverage -p 8 --normalizeUsing RPKM --binSize 10 -b *.sort.bam -o *.bw # the *bw file was loaded to the IGV for visualization

7. **Calculation of RPKM**

this script was used to calculate the RPKM values for all the samples in this study.

input data is the read count matirx

the number 80.89 was calculated from1000 / library size (M)

column 6 ($6) is gene length

```
cat *count.txt | awk '{print $1,$7*80.89/$6}' OFS="\t" | sort -k1,1 -k2,2n > *RPKM.txt
```

8. Calculation of TPM

```
# this script was used to calculate the TPM values for all the wounding-treated samples;
# log2TPM was used to perform correlation analysis in Figure 1A, Figure S2B-D.
# input data is the read count matrix
Count<-read.csv("*count_matrix.csv",head=T,stringsAsFactors = F)
kb <- Count$Length/ 1000 # Length is the gene length
countdata <- Count[,3:8] # column 1 is the gene ID; column 2 is the gene length
rpk <- countdata / kb
tpm <- data.frame(t(t(rpk)/colSums(rpk) * 1000000))
head(tpm)
tpm$gene=Count$Geneid # add the gene ID to the TPM file
write.csv(tpm,file="*TPM.csv",row.names = F)
```

9. Calculation of Pearson correlation coefficient

```
# input data is the log2TPM matrix for Figure 1A, Figure S2B-D
# input data is the log2FC matrix for Figure 1B-F, Figure 2B and C, Figure S4, Figure S5
# fold change (FC) is obtained from section 10
data=read.table("log2 TPM(FC).txt", header=T, row.names=1, sep="\t")
head(data)
cor_matr=cor(data)
head(cor_matr)
write.table(cor_matr,"coefficient_matrix.txt",sep="\t")
```

10. Differential expression analysis by DESeq2 (R package)

```
# this script was used to call differentially expressed genes
# input data is the read count matrix
m=read.delim("count_matrix.txt", head=T)
colnames(m)=c("Gene_ID","control_1","control_2","control_3","stress_1","stress_2","stress_3")
rownames(m) <-m[,1]
count_data<-m[,2:7] # column 1 is the gene ID
condition<-factor(c(rep("control",3), rep("stress",3)), levels = c("control","stress")) # “3”
represents the number of biological replicates for control and stress samples
col_data<-data.frame(row.names=colnames(count_data), condition)
library("DESeq2")
dds <- DESeqDataSetFromMatrix(countData=count_data,colData=col_data,design= ~ condition)
dds<-DESeq(dds)
res<-results(dds)
summary(res)
resdata=merge(as.data.frame(res),as.data.frame(counts(dds,normalize=TRUE)),by="row.names",s
ort=FALSE)
write.csv(resdata,file="diff_res.csv",row.names=F)
```

11. Calculation of translation efficiency (TE) changes using DESeq2

```
# translational efficiency (TE) of each gene is defined as the ratio of RPF to total mRNA;
# under stress conditions, changes in translational efficiencies (TE) can be calculated by ratios of ratios for two conditions: (Ribo_stress/mRNA_stress)/(Ribo_control/mRNA_control)
# input data is the read count matrix
m=read.delim("count_matrix.txt",head=T)
colnames(m)=c("Gene_ID","RPF_control_1","RPF_control_2","RPF_control_3","RPF_stress_1","RPF_stress_2","RPF_stress_3","RNA_control_1","RNA_control_2","RNA_control_3","RNA_stress_1","RNA_stress_2","RNA_stress_3")
rownames(m) <-m[,1]
count_data<-m[,2:13] # column 1 is the gene ID
count_data<-round(as.matrix(count_data))
condition<-factor(c(rep("control",3), rep("stress",3), rep("control",3), rep("stress",3)), levels = c("control","stress")) # “3” represents the number of biological replicates for control and stress samples
col_data<-data.frame(assay=factor(rep(c("Ribo", "mRNA"), each=4)),condition=condition,row.names=colnames(count_data))
write.table(col_data,file="coldata.txt",row.names=F,quote=F,sep="\t")
library("DESeq2")
dds<-DESeqDataSetFromMatrix(countData=as.matrix(count_data[,row.names(col_data)]),colData=col_data,design=~ assay+condition+assay:condition)
model.matrix(~ assay+condition+assay:condition, col_data)
dds <-DESeq(dds, test="LRT", reduced=~ assay+condition)
res<- DESeq2::results(dds)
head(res[order(res$padj),],4)
write.table(res, file="TE.txt",quote=F, col.names=NA, sep="\t")
resdata=merge(as.data.frame(res),as.data.frame(counts(dds,normalize=TRUE)),by="row.names",sort=FALSE)
write.csv(resdata,file="TE.csv",row.names = F)
```

12. GO analysis

```
x = read.table("genelist.txt",header=F)    # input data is the gene ID of differentially expressed genes
head(x)
x = as.vector(as.matrix(x[,1]))
ego_ALL <-enrichGO(x, OrgDb="org.At.tair.db", keyType="TAIR", ont="ALL", pvalueCutoff=0.05, pAdjustMethod="BH", qvalueCutoff=0.1, readable=T)
write.csv(ego_ALL, file="GO_ALL_TAIR.csv", quote=F)
```

13. Meta-analysis

the following code allows to reproduce metagene plots in Figure S2G and Figure S3

# 100 bp upstream/downstream of the start/stop codon were extracted and split into 1-bp bin. Thus, for each gene, there are 200 bins around the start or stop codon, respectively

```
# extract the annotated start or stop codons from Araport11 annotation
awk '{if($3=="start_codon")print $0}' OFS="\t" araport11.gtf > start_codon.gtf
awk '{if($3=="stop_codon")print $0}' OFS="\t" araport11.gtf > stop_codon.gtf

# convert the gtf file to bed format
cut -f 9 start_codon.gtf | cut -d ";" -f 2 | sed 's/ transcript_id//g' | paste - start_codon.gtf | awk '{print $2,$5-1,$6,$1,1,$8}' OFS="\t" | sort -k1,1 -k2,2n > start_codon.bed
cut -f 9 stop_codon.gtf | cut -d ";" -f 2 | sed 's/ transcript_id//g' | paste - stop_codon.gtf | awk '{print $2,$5-1,$6,$1,1,$8}' OFS="\t" | sort -k1,1 -k2,2n > stop_codon.bed

# extend by 100 bp upstream and downstream of the start/stop codon
awk '{if($6=="+"){$7=$2-100;$8=$2+100;print$1,$7,$8,$4,$5,$6}else{$7=$3-100;$8=$3+100;print$1,$7,$8,$4,$5,$6}}' OFS="\t" start_codon.bed > start_codon±100bp.bed
awk '{if($6=="+")print $0}' OFS="\t" start_codon±100bp.bed > start_codon.plus.bed
awk '{if($6=="-")print $0}' OFS="\t" start_codon±100bp.bed > start_codon.minus.bed
awk '{if($6=="+"){$7=$2-100;$8=$2+100;print$1,$7,$8,$4,$5,$6}else{$7=$3-100;$8=$3+100;print$1,$7,$8,$4,$5,$6}}' OFS="\t" stop_codon.bed > stop_codon±100bp.bed
awk '{if($6=="+")print $0}' OFS="\t" stop_codon±100bp.bed > stop_codon.plus.bed
awk '{if($6=="-")print $0}' OFS="\t" stop_codon±100bp.bed > stop_codon.minus.bed

# make 1-bp bins
bedtools makewindows -b start_codon.plus.bed -w 1 -i srcwinnum | tr "_" "\t" | awk 'BEGIN{OFS="\t"}{$6="+";print $0}' > start_codon.plus.bin.bed
bedtools makewindows -b start_codon.minus.bed -w 1 -i srcwinnum | tr "_" "\t" | awk 'BEGIN{OFS="\t"}{$6="-";print $0}' > start_codon.minus.bin.bed
awk '{print $5}' start_codon.minus.bin.bed | tac > minus.start_codon.order
paste minus.start_codon.order start_codon.minus.bin.bed | awk '{print $2,$3,$4,$5,$1,$7}' OFS="\t" > start_codon.minus.bin2.bed
cat start_codon.plus.bin.bed start_codon.minus.bin2.bed | sort -k1,1 -k2,2n > start_codon.1bpbin.bed

bedtools makewindows -b stop_codon.plus.bed -w 1 -i srcwinnum | tr "_" "\t" | awk 'BEGIN{OFS="\t"}{$6="+";print $0}' > stop_codon.plus.bin.bed
bedtools makewindows -b stop_codon.minus.bed -w 1 -i srcwinnum | tr "_" "\t" | awk 'BEGIN{OFS="\t"}{$6="-";print $0}' > stop_codon.minus.bin.bed
```

```
awk '{print $5}' stop_codon.minus.bin.bed | tac > minus.stop_codon.order
paste minus.stop_codon.order stop_codon.minus.bin.bed | awk '{print $2,$3,$4,$5,$1,$7}'
OFS="\t" > stop_codon.minus.bin2.bed
cat stop_codon.plus.bin.bed stop_codon.minus.bin2.bed | sort -k1,1 -k2,2n >
stop_codon.1bpbin.bed

sed 's/"//g' start_codon.1bpbin.bed > start_codon.1bpbin_1.bed
sort -k1,1 -k4,4 start_codon.1bpbin_1.bed > start_codon.1bpbin_2.bed
sed 's/"//g' stop_codon.1bpbin.bed > stop_codon.1bpbin_1.bed
sort -k1,1 -k4,4 stop_codon.1bpbin_1.bed > stop_codon.1bpbin_2.bed

# The first base of the 5′ end was extracted from the uniquely mapped reads (*.bam) and
then conveted to bed format
# for pair-end mapping
samtools sort -n *.bam > *.sortbyname.bam
bamToBed -i *.sortbyname.bam -bedpe -mate1 > *.sortbyname.bedpe
awk '{if($9=="+"){print $1,$2,$2+1,$7,$8,$9}else{print $1,$3-1,$3,$7,$8,$9}}' OFS="\t"
*.sortbyname.bedpe > *.onebase.bed

# for single-end mapping
bamToBed -i *.bam | awk '{if($6=="+"){$3=$2+1;print $0}else{$2=$3-1;print $0}}' OFS="\t" >
*.onebase.bed

# Calculate the read coverage (RPKM) around the start or stop codon for reads mapped to
sense or antisense strand, respectively
# the number 89.58 was calculated from 1000 / library size (M)
# column 9 ($9) is gene length
coverageBed -a start_codon.1bpbin_2.bed -b *onebase.bed -s | awk '{print $4,$5,$7*89.58/$9}'
OFS="\t" | sort -k1,1 -k2,2n > *start.sense.txt
coverageBed -a stop_codon.1bpbin_2.bed -b *onebase.bed -s | awk '{print $4,$5,$7*89.58/$9}'
OFS="\t" | sort -k1,1 -k2,2n > *stop.sense.txt

coverageBed -a start_codon.1bpbin_2.bed -b *onebase.bed -S | awk '{print $4,$5,$7*89.58/$9}'
OFS="\t" | sort -k1,1 -k2,2n > *start.antisense.txt
coverageBed -a stop_codon.1bpbin_2.bed -b *onebase.bed -S | awk '{print $4,$5,$7*89.58/$9}'
OFS="\t" | sort -k1,1 -k2,2n > *stop.antisense.txt

# Prepare the data for plotting
require(ggplot2)
metaplot_1<-function(a){
  i=floor(length(a[,1])*0.01)
  j=ceiling(length(a[,1])*0.99)
  a.sort=apply(a,2,sort)
  a.trim=a.sort[i:j,]
```

```
    a.mean=apply(a.trim,2,mean)
    a.se=apply(a.trim,2,sd)/sqrt(length(a.trim[,1]))
    a.d=data.frame(x=seq(-99,100,1),mean=a.mean,se=a.se) }

metaplot_2<-function(a){
    i=floor(length(a[,1])*0.01)
    j=ceiling(length(a[,1])*0.99)
    a.sort=apply(a,2,sort)
    a.trim=a.sort[i:j,]
    a.mean=apply(a.trim,2,mean)
    a.se=apply(a.trim,2,sd)/sqrt(length(a.trim[,1]))
    a.d=data.frame(x=seq(-99,100,1),mean=a.mean*(-1),se=a.se) }

start.sense<-read.table("*start.sense.txt", sep="\t")
start.m<-matrix(start[,3],ncol=200,byrow=T)
rownames(start.m)<-unique(start[,1])
write.table(start.m,file="*start.sense.m.txt",sep="\t", row.names=F)
start.sen.d<-metaplot_1(start.m)
start.sen.d$pos="start"
start.sen.d$sample_strand="sense"
write.table(start.sen.d,file="*start.sense.data.txt",sep="\t", row.names=F)

Start.anti<-read.table("*start.antisense.txt", sep="\t")
start.m<-matrix(start [,3],ncol=200,byrow=T)
rownames(start.m)<-unique(start[,1])
write.table(start.m,file="*start.anti.m.txt",sep="\t", row.names=F)
start.anti.d<-metaplot_2(start.m)
start.anti.d$pos=""start "
start.anti.d$sample_strand ="anti"
write.table(start.anti.d,file="*start.anti.data.txt",sep="\t", row.names=F)


stop.sense<-read.table("*stop.sense.txt", sep="\t")
stop.m<-matrix(stop[,3],ncol=200,byrow=T)
rownames(stop.m)<-unique(stop[,1])
write.table(stop.m,file="*stop.sense.m.txt",sep="\t", row.names=F)
stop.sen.d<-metaplot_1(stop.m)
stop.sen.d$pos=""stop"
stop.sen.d$sample_strand="sense"
write.table(stop.sen.d,file="*stop.sen.data.txt",sep="\t", row.names=F)

stop.anti<-read.table("*stop.anti.txt", sep="\t")
stop.m<-matrix(stop[,3],ncol=200,byrow=T)
rownames(stop.m)<-unique(stop[,1])
```

```
write.table(stop.m,file="*stop.anti.m.txt",sep="\t", row.names=F)
stop.anti.d<-metaplot_2(stop.m)
stop.anti.d$pos="stop"
stop.anti.d$sample_strand ="anti"
write.table(stop.anti.d,file="*stop.anti.data.txt",sep="\t", row.names=F)

data<-rbind(start.sen.d, start.anti.d, stop.sen.d, stop.anti.d)
write.table(data,file="meta_data_for_plotting.txt",sep="\t", row.names=F)

# Plot the results of meta-analysis
data<-read.csv("meta_data_for_plotting.csv",head=T,stringsAsFactors = F)
head(data)
pdf("metaplot.pdf",width = 10, height = 2.8)
ggplot(data,aes(x=x,y=mean,colour=sample_strand))+
scale_color_manual(limits =
c("control1_sense","control2_sense","control3_sense","stress1_sense","stress2_sense","stress3_se
nse","control1_anti","control2_anti","control3_anti","stress1_anti","stress2_anti","stress3_anti"),v
alues=c("black","grey","red","orange","blue","darkgreen","black","grey","red","orange","blue","d
arkgreen")) +
geom_linerange(aes(x,ymin=mean-se,ymax=mean+se))+
geom_line(size=0.3)+
geom_hline(yintercept=0,colour="black",linetype="dashed",size=0.2)+
geom_vline(xintercept = 0,colour="black",linetype="dashed",size=0.2)+
labs(y = "Reads density",x= "Distance from
start_codon/stop_codon")+guides(size=guide_legend())+
theme(panel.grid.minor=element_blank(),panel.background=element_blank(),
panel.border=element_rect(fill=NA))+
facet_grid(~pos)
dev.off()
```
